# Contaminants of Emerging Concern: From the Detection to Their Effects on Human Health

**DOI:** 10.1155/2016/3159385

**Published:** 2016-06-20

**Authors:** Fernando Barbosa Junior, Andres Campiglia, Bruno Rocha, Daniel Cyr

**Affiliations:** ^1^Departamento de Análises Clínicas, Toxicológicas e Bromatológicas, Faculdade de Ciências Farmacêuticas de Ribeirão Preto, Universidade de São Paulo, 14040903 Ribeirão Preto, SP, Brazil; ^2^Department of Chemistry, University of Central Florida, Orlando, FL, USA; ^3^INRS-Institut Armand Frappier, Université du Québec, 531 boulevard des Prairies, Laval, QC, Canada H7V 1B7

In the past years with the advance of analytical instrumentation/methods, some chemicals are being discovered in water and other environmental matrices that previously had not been noticed or are being detected at levels that may be diverse more than expected. These are often generally referred to as “contaminants of emerging concern” because the potential risk to human health and the environment associated with their presence, frequency of occurrence, or source may not be known. Some of these compounds include household and industrial chemicals such as same retardants, plasticizers, detergent compounds, pharmaceutical and personal care products, fragrances, antimicrobial cleaning agents, nanomaterials, and some toxic elements [1–5].

This special issue affords the opportunity to bring together the results of 5 research articles and 1 review paper covering several aspects related to this topic.

An overview of emerging contaminants and associated human health effects was presented by M. Lei et al. The authors have summarized the conclusions of the comprehensive epidemic literature and representative case reports relevant to emerging contaminants and the human body to address concerns about potential harmful health effects in the general population. They concluded that the current evidence is not conclusive and comprehensive, suggesting prospective cohort studies in the future to evaluate the associations between human health outcomes and emerging environmental contaminants.

W.-H. Yu et al. evaluated the expression and function of Oat1 and Oat3 after administering arsenic and mercury containing traditional Chinese medicine (realgar and cinnabar)* in vivo* in mice. The authors have found that the traditional Chinese medicine investigated is probably related to kidney damage through inhibiting several members of the organic anion transporters (such as OAT1 and OAT3).

Q. Liu et al. evaluated whether there were miRNA and mRNA aberrant expression profiles and potential role in malignant transformation of 16HBE induced by cadmium. Their results provided a link for the miRNA-mRNA integrated network and implied the role of novel miRNAs in malignant transformation of 16HBE induced by cadmium.

N. Sarker et al. determined the levels of various metals in three different floral honeys from Bangladesh. Several physical parameters were also determined. According to the authors, honeys from Bangladesh are of good quality and the tested parameters met the requirements set by the International Honey Commission. The heavy metal concentrations were also within the limits, indicating their purity. Moreover, the higher concentrations of Fe in the investigated honeys signify that these honeys are a good source of these elements, which is very important for the human diet.

Q. Hu et al. concluded that mycotoxins from mycoinsecticides have limited ways to enter environments. Moreover, according to the authors the risks of mycotoxins from mycoinsecticides contaminating foods are likely controllable.

I. Macharia investigates the determinants of pesticide-related cost of illness (COI) and acute symptoms, using a balanced panel of 363 farmers interviewed from seven major vegetable producing districts of Kenya. The author observed that the personal protective equipment (PPE), education level, record keeping, and geographical location considerably determined health impairments. Moreover, encouraging the proper use of PPE and record keeping of pesticide use could greatly reduce poisoning cases and COI.
